# Three-Dimensional Motion Capture Data of a Movement Screen from 183 Athletes

**DOI:** 10.1038/s41597-023-02082-6

**Published:** 2023-04-24

**Authors:** Xiong Zhao, Gwyneth Ross, Brittany Dowling, Ryan B. Graham

**Affiliations:** 1 School of Human Kinetics, Faculty of Health Sciences, University of Ottawa, Ottawa, Ontario Canada; 2 Motus Global, LLC., Rockville Centre, New York, USA

**Keywords:** Risk factors, Rehabilitation

## Abstract

Movement screens are widely used to identify aberrant movement patterns in hopes of decreasing risk of injury, identifying talent, and/or improving performance. Motion capture data can provide quantitative, objective feedback regarding movement patterns. The dataset contains three-dimensional (3D) motion capture data of 183 athletes performing mobility tests (ankle, back bend, crossover adduction, crossover rotation, elbows, head, hip turn, scorpion, shoulder abduction, shoulder azimuth, shoulder rotation, side bends, side lunges and trunk rotation) and stability tests (drop jump, hop down, L-cut, lunge, rotary stability, step down and T-balance) bilaterally (where applicable), the athletes’ injury history, and demographics. All data were collected at 120 Hz or 480 Hz using an 8-camera Raptor-E motion capture system with 45 passive reflective markers. A total of 5,493 trials were pre-processed and included in .c3d and .mat formats. This dataset will enable researchers and end users to explore movement patterns of athletes of varying demographics from different sports and competition levels; develop objective movement assessment tools; and gain new insights into the relationships between movement patterns and injury.

## Background & Summary

Movement screens are widely used across many disciplines including in ergonomic, clinical, and athletic settings to identify aberrant movement patterns in hopes of decreasing risk of injury, identifying talent, and/or improving performance^1–7^. Researchers have previously shown that young athletes who perform better on non-sport specific movement batteries are more likely in the future to compete at a more elite level compared to athletes that perform more poorly on the movement batteries^8–11^. Performance on a non-sport-specific movement battery was a better indicator for future sport performance than subjective coach rankings^11^, anthropometrics^10,11^, or current competition rankings^11^. The Functional Movement Screen (FMS; Functional Movement Systems, Chatham, VA, USA) is a commonly used quantitative movement screen amongst coaches and clinicians.

Most commonly, during a movement screen, an individual’s movement is evaluated based on visual appraisal^5^; however, there is agreement within the literature that inter- and intra-rater reliability of these subjective movement screens are poor^12–14^. Limitations of these tests include: (1) the difference between scores needs to be large enough for the human eye to detect; (2) the scores are ordinal and discrete allowing for only a few scores to be available (e.g., FMS only has 4 options, 0–3), which may not be sensitive enough to capture variability between athletes nor dysfunction; (3) individuals are able to increase their scores when aware of the scoring criteria; and (4) task-specific criteria are not linked to any known biomechanical or ergonomic risk factors^15,16^.

Motion capture systems can detect differences in movements much smaller than can be seen and processed by a human observer, and give measures that can provide coaches, clinicians, and athletes with quantitative, objective feedback. However, to gain meaningful insight, large datasets are needed, which are often not accessible to researchers. With such a large dataset, more advanced analyses involving pattern recognition and machine learning to classify or predict movement can be applied. Objective movement quality assessments can provide coaches and therapists with reliable quantitative results and help them with decisions on player performance and safety. Various machine learning algorithms have been used in the biomechanics field to recognize movement patterns^17^, analyse sport techniques^18,19^, and objectively differentiate movement patterns between elite and novice athletes based on whole-body kinematics^20^.

To date, there is only one publicly available dataset containing movement screening (i.e., FMS) data. The data were collected using two Azure Kinect depth sensors and are focused on the development of an automatic movement scoring tool^21^. In comparison, our dataset focuses on exploring the potential relationships between movement quality and injury risk, differences between sports/competition level, and the effects of demographics on movement patterns. The dataset contains more participants and a greater range of movements; was collected using the current gold standard in biomechanics for motion capture, a passive, optical motion capture system; and provides a 10-year injury history of each participant.

In this paper, we present a dataset of 3D kinematic data of 183 athletes that was collected to assess movement competency of the athletes and to investigate the development of objective movement screening tools. The athletes ranged in age from 9 to 36 years old and competed in different sports and competition levels.

## Methods

All data were collected by Motus Global (Rockville Centre, New York, USA), a for-profit company whose mission was to develop solutions that enhanced human performance, monitor rehabilitation, and provide insights to reduce the risk of injuries through motion capture technology, between 2012–2016. During collection, athletes performed Motus Global’s proprietary movement screening protocol.

### Participants

Data were collected on 183 (153 males, 30 females) athletes competing in one of eight sports (i.e., baseball, basketball, football, golf, lacrosse, soccer, tennis, and track and field) and ranging in competition level from youth to professional (e.g., NFL, NBA, MLB, FIFA), with athletes competing at the youth, middle school, high school, postgraduate, college/draft, and professional level. The average age, height, and weight was 20.35 ± 4.20 years, 183.08 ± 15.33 cm, and 84.51 ± 22.51 kg, respectively. When broken down by sex, males had an average age, height and weight of 21 ± 4.12 years, 186.21 ± 14.72 cm, and 89.14 ± 21.46 kg, respectively and females had an average age, height, and weight of 17 ± 2.73 years, 167.16 ± 5.02 cm, and 60.93 ± 8.82 kg, respectively. Prior to data collection, each athlete signed an informed consent form providing permission for their data to be used for future research. If the athlete was under the age of consent, permission was granted by their legal guardian/next of kin. The detailed ID coded participant information, as well as their injury history, is provided (‘Subject Log.xlsx’ in the folder ‘Subject Info’). The Health Sciences Research Ethics Board at the University of Ottawa approved the secondary use of the data for research purposes (file no: H-08-18-1085).

### Equipment

**Fig. 1:**
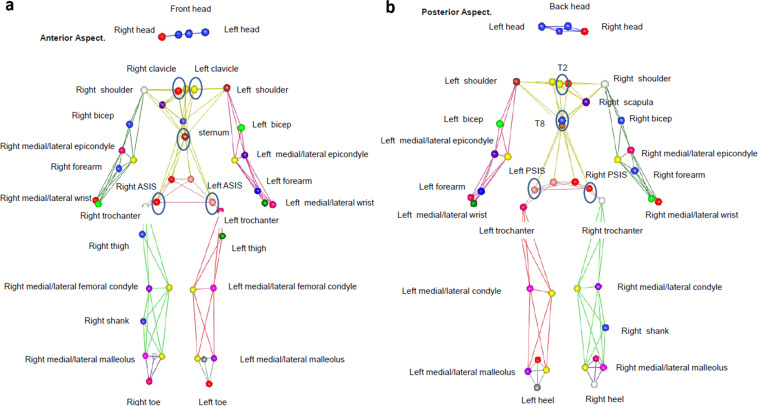
Marker names and placement from anterior (**a**) and posterior (**b**) views. The circled markers are the ones that are only shown on the posterior or anterior view and not both.

To capture whole-body kinematics, athletes were outfitted with 45 passive, reflective markers (B&L Engineering, Santa Ana, CA, USA). Thirty-seven of the markers were placed on anatomical landmarks to define the head, trunk, upper arms, forearms, pelvis, thighs, shanks, and feet segments. These markers were placed on the front, back, left and right side of the head; left and right clavicle; left and right acromion process; xyphoid process of the sternum; 2^nd^ and 8^th^ thoracic vertebrae; left and right anterior superior iliac spine (ASIS); left and right posterior superior iliac spine (PSIS); left and right medial and lateral epicondyles of the humerus; left and right ulnar and radial styloid processes; left and right greater trochanters; left and right medial and lateral condyles of the femur; left and right medial and lateral malleoli; left and right heel; and left and right head of the 2^nd^ metatarsal. Eight additional tracking markers, to assist with tracking segments and to help identify the left and right limbs, were placed bilaterally on the thighs, forearms, and biceps, and unilaterally on the right shank and scapula (Fig. 1). All data were collected at 120 Hz or 480 Hz using an 8-camera Raptor-E motion capture system (Motion Analysis Corporation, Santa Rosa, CA, USA).

### Protocol

Upon arriving at the Motus Global laboratory, athletes read and signed the informed consent form, had their height (with shoes) and weight taken, and provided details regarding their injury history from the previous ten years. Injury data included the number and location of injuries.  Athletes were then outfitted with the markers described above. After being outfitted with the markers, the athlete performed a static calibration trial. In the static calibration trial, the athlete stood in the ‘motorcycle’ position with their feet shoulder-width apart, toes pointing straight forward, arms abducted 90° at the shoulder, elbows bent at 90°, and wrists in line with the forearm.

Following calibration, athletes performed a 21-movement, proprietary protocol that was designed to assess the athletes’ range of motion (ROM) at each joint and overall stability, power, and balance. Mobility tests included: ankle, back bend, crossover adduction, crossover rotation, elbows, head, hip turn, scorpion, shoulder abduction, shoulder azimuth, shoulder rotation, side bends, side lunges and trunk rotation, and the stability tests included: drop jump, hop down, L-cut, lunge, rotary stability, step down and T-balance (Supplementary File 1). With the exception of the drop jump, backbend, head, side bends, and trunk rotation, all movements were performed bilaterally. Within the dataset, there are 30 trials in total; six movements were performed unilaterally (drop jump, backbend, head, side bends and trunk rotation), six movements were performed bilaterally within the same trial (ankle, elbows, lunge, shoulder rotation, shoulder azimuth, and shoulder abduction), and the remaining nine movements were performed bilaterally in separate trials, resulting in 18 trials. For each movement, the athletes performed the movement until they believed they had completed the task to the best of their ability. Only their best trial was kept and analysed.

### Data pre-processing

The kinematic data were labelled, cleaned and gap-filled in Cortex (Motion Analysis, Santa Rosa, CA, USA). Once labelled and clean, data were exported to Visual3D (C-Motion, Inc., Germantown, MD, USA), where a full-body model was developed based on the static calibration trial. The model was then applied to all motion trials after the marker trajectories were filtered using a low-pass, 2^nd^ order Butterworth filter with a cut-off frequency of 10 Hz. 3D joint centre (JC) positional data of the head, wrists, elbows, shoulders, distal end of the feet, ankles, knees, and hips; 3D centre of gravity positional data for the trunk, head and pelvis; 3D positional raw data for the left and right heel, T2, T8, sternum, and the back, front, and sides of the head were calculated. 3D joint angles (JA) of the neck, upper back, lower back, shoulders, elbows, hips, knees and ankles, and 3D segment velocity (SV) and acceleration of the head, pelvis, trunk, forearms, upper-arms, feet, shank and thighs were calculated and extracted. Both processed and raw files were exported from Visual3D as three .mat files (JC, JA, and SV) for each participant, which can be accessed using Matlab (The Mathworks, Inc., Natick, MA, USA). For the processed files, the data were filtered with a low-pass Butterworth filter with a cut-off frequency of 10 Hz. All data in .mat files are stored at their original sampling frequency (120 Hz or 480 Hz).

## Data Records

**Fig. 2:**
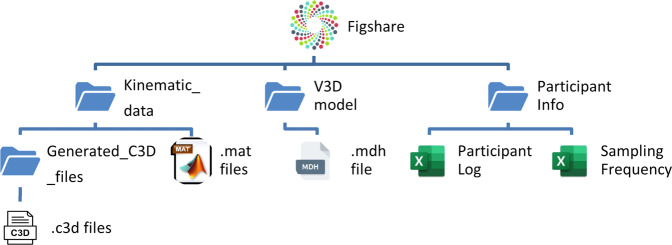
Hierarchical folder structure of the dataset.

This dataset is available on Figshare^22^. In this dataset, there are three folders in total (*Participant Info*, *V3D model*, and *Kinematic_Data*). Two excel files named *Subject Log* and *Sampling Frequency* can be found in the *Participant Info* folder. In the *V3D model* folder, there is a Visual 3D model template file (‘SMBL_OS_Model.mdh’) that can be applied to the raw .c3d files in Visual3D (C-Motion, Inc., Germantown, MD, USA) to build a full-body biomechanical model for each participant. Each athlete has a number-coded catalogue which contains a sub-folder titled *Generated_C3D_files* and 16 .mat files. The dataset is stored in a hierarchical folder structure (Fig. 2).

The excel file, *Subject Log*, provides de-identified demographic data (gender, age, height, and weight), sports data (sport and level), and injury information for all injuries that resulted in missing at least 1 game/competition from the past 10 years (number and loation of injuries) for each participant. The other excel file, *Sampling Frequency*, records the sampling frequency (120 or 480 Hz) that the data were captured at for all tasks for that participant. In each *Generated_C3D_files* folder, there are 31 trials (1 calibration trial and 30 motion trials) saved as .c3d. The following naming convention was used:

- Calib1.c3d
- PPID_TASK_##.c3d

where *Calib1.c3d* is universally named for the calibration trial in each participant’s folder, *PPID* represents the participant’s four-digit ID, *TASK* represents the name of the aforementioned movement tasks, and *##* indicates an optional two-digit note.

The three .mat files, *JA_PPID.mat*, *JC_PPID.mat*, and *SV_PPID.mat*, contain joint angles (JA) in degrees, 3D coordinates of the joint centres (JC) in meters, and segment velocities (degrees/second) and acceleration (degrees/second²) (SV), respectively. Participant’s ID is denoted by *PPID*. In *JA_PPID*, both raw and processed joint angles of each segment are stored in a 30 × 1 cell. The variable *FILE_NAME* contains the file name of each motion trial and the order of which the file names match that of the joint angle data in each cell. Within each of the cells, there is an [n (frames) × 3 (three dimensions)] matrix containing the 3D joint angles of the movement task. Variables contained in *JA_PPID.mat* can be divided into two categories:

1. Joint angles:
  Neck: NECK_O, NECK_P
  Lower Back: TRNK_O, TRNK_P
  Shoulder: L_SHLDR_O, L_SHLDR_P, R_SHLDR_O, R_SHLDR_P, L_ELB_O, L_ELB_P, R_ELB_O, R_ELB_P
  Hip: L_HIP_O, L_HIP_P, R_HIP_O, R_HIP_P
  Knee: L_KN_O, L_KN_P, R_KN_O, R_KN_P
  Ankle: L_ANK_O, L_ANK_P, R_ANK_O, R_ANK_P
2. Segment angles relative to lab origin (global coordinate system):
  Pelvis: P_O, P_P
  Upper back: THRX_O, THRX_P
  Upper arm: L_UA_O, L_UA_P, R_UA_O, R_UA_P
  Forearm: L_FA_O, L_FA_P, R_FA_O, R_FA_P
  Thigh: L_T_O, L_T_P, R_T_O, R_T_P
  Shank: L_S_O, L_S_P, R_S_O, R_S_P
  Foot: L_F_O, L_F_P, R_F_O, R_F_P

where ‘L’, ‘R’, ‘O’ and ‘P’ stand for left, right, original, and processed (filtered), respectively. All joint angles were calculated using the provided Visual3D model, which does not have any IK constraints, and were extracted following an X-Y-Z Cardan sequence, where flexion/extension is around the x-axis, adduction/abduction is around the y-axis, and the rotation is around the z-axis.

In *JC_PPID.mat*, 3D positional data of each joint centre were stored in a 30 × 1 cell. The variable *FILE_NAME* contains the file name of each motion trial and the order in which the file names correspond to that of the positional data in each cell. Inside of each cell, an [n (frames) × 3 (dimensions)] matrix was used to save the positional data of the movement. The following variables were calculated and stored in *JC_PPID.mat*:

1. Marker Data: FHEAD, BHEAD, LHEAD, RHEAD, STERN, T2POS, T8POS, TRNKP, MID_ASIS, MID_PSIS, LHEEL, RHEEL
2. Centre of Gravity Data: HEADCOG, TRNKCOG, PLVCOG
3. Joint Centre Data: LUAD, LUAP, RUAD, RUAP, LFAD, LFAP, RFAD, RFAP, LTHD, LTHP, RTHD, RTHP, LSHD, LSHP, RSHD, RSHP, LFTD, LFTP, RFTD, RFTP

where Marker Data are the positional data for the front, back, left and right side of the head, sternum, T2, T8, trunk (upper), mid-point between the left and right anterior and posterior superior iliac spines, and left and right heel, respectively; Centre of Gravity Data are the 3D centre of gravity positional data of the head, trunk and pelvis, respectively; and Joint Centre Data contains the 3D positional data of the proximal and distal ends of the upper arms, forearms, thighs, shanks, and feet, respectively. ‘L’ or ‘R’ at the beginning of each variable name denotes the left or right side and ‘D’ or ‘P’ at the end of each variable represents the distal or proximal end of the segment.

In *SV_PPID.mat*, 3D orientation and acceleration data of each segment were stored in a 30 × 1 cell. The order of the data is the same as the file name in variable *FILE_NAME*. Similarly, within each cell, an [n (frames) × 3 (three dimensions)] matrix was used to store the data of a test movement. The following variables of each segment were saved in *SV_PPID*:

**_angacc, **_angpos, **_angvel, **_segpos, **_segvel

where ‘**’ represents the different body-parts analysed: head, pelvis, thorax, and the left or right side of the upper-arm, forearm, thigh, shank, and foot. ‘angacc’, ‘angpos’, ‘angvel’, ‘segpos’, and ‘segvel’ stand for segment angular acceleration, segment orientation, angular velocity, segment centre of mass (COM), and segment COM velocity relative to lab, respectively.

The other 13 .mat files (startstop_PPID_TASK_final.mat) store the event indices of the seven stability tasks (drop jump, hop down, L-cut, lunge, rotary stability, step down and T-balance) that were automatically selected based on criteria described in Supplementary File 1 and then visually verified by a trained researcher. In *‘startstop_PPID_LL_final.mat’*, *‘startstop_PPID_LR_final.mat’*, *‘startstop_PPID_RSL_final.mat’*, *‘startstop_PPID_RSR_final.mat’*, *‘startstop_PPID_SDL_final.mat’*, *‘startstop_PPID_SDR_final.mat’*, *‘startstop_PPID_TBL_final.mat’*, and *‘startstop_PPID_TBR_final.mat’*, there are two variables, *start* and *stop*, containing the frame number of the start and end of the test, respectively. In *‘startstop_PPID_DJ_final.mat’*, apart from the *start* and *stop, jumpdown* and *takeoff* were added to indicate the time of the participant jumping off the platform and taking off after landing on the floor, respectively. In *‘startstop_PPID_HDL_final.mat’* and ‘*startstop_PPID_HDR_final.mat’*, in addition to the *start* and *stop*, three other variables, *zero*, *min*, and *max*, were created to identify the moment the participant jumped off the elevated platform, touched the ground, and took off from the ground, respectively. In *‘startstop_PPID_LHL_final.mat’* and ‘*startstop_PPID_LHR_final.mat’*, *maxheight*, *landing*, and *stop* represent the frame number of the maximal height, the landing, and end of the jump, respectively. Event indices of each trial represent their original frame numbers. In order, LL, LR, RSL, RSR, SDL, SDR, TBL, TBR, DJ, HDL, HDR, LHL and LHR stand for Lunge Left, Lunge Right, Rotary Stability Left, Rotary Stability Right, Step Down Left, Step Down Right, T-Balance Left, T-Balance Right, Drop Jump, Hop Down Left, Hop Down Right, L-Hop (L-cut) Left and L-Hop (L-cut) Right.

In total, the dataset contains 5,676 motion capture trials (including calibration trials) saved in .c3d format (participant 1424’s Scorpion and Ankle were split into left and right trials and participant 1913’s Ankle was split into left and right trials), and 2928 (183 × 16) .mat files containing kinematic/indexing data.

## Technical Validation

All raw marker trajectories are expected to be accurate within 0.2 mm to 2 mm based on previous research validating the optical motion capture system^23,24^. Marker placement was performed by experienced researchers. The parameters of the motion capture system were adjusted for optimal marker visibility and noise reduction and the capture volume calibration (camera calibration and volume origin setting) was performed before each session.

After labelling the 3D trajectories of all reflective markers in each motion trial, gap filling was done using a spline in the Cortex software when there was a short period of missing marker trajectory during the motion trial. Trials with missing marker(s) or had a large gap were not included in the dataset. This process was executed by well-trained researchers.

For the validation, a whole body-model was built in Visual3D (C-Motion, Inc., Germantown, MD, USA) using the default *‘V3D 6 DOF’* algorithm (i.e., without IK constraints). To include IK constraints, one will need to modify the provided Visual3D model in Visual3D by entering the desired IK constraints and switching the constraints from ‘*V3D 6 DOF*’ to ‘*Inverse Kinematics*’ in the ‘*Choose Algorithm for Computing Pose*’ drop down menu under the ‘*IK Constraints*’ tab.

**Fig. 3:**
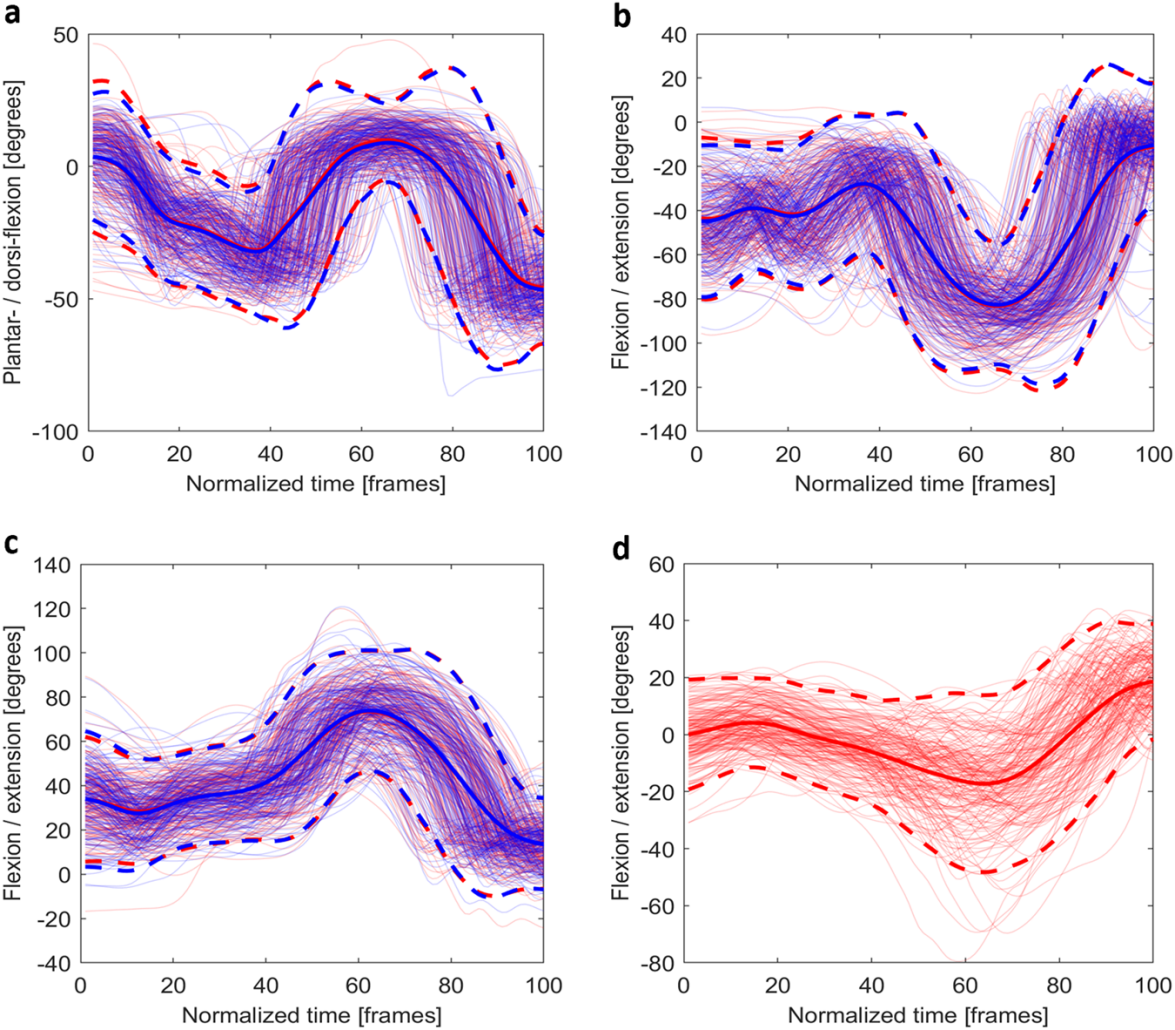
The time normalised joint angles for (**a**) the plantar-/dorsi-flexion (+) of the ankle, (**b**) the flexion/extension (+) of the knee, (**c**) the flexion (+) /extension of the hip, and (**d**) the flexion/extension (+) of the trunk (red) during the *drop jump*. For bilateral joints, the left (blue) and right (red) are shown. Mean joint angles (solid) and plus/minus two standard deviations (dash) are plotted using opaque lines with the same colour of the joint angles for that side.

**Fig. 4:**
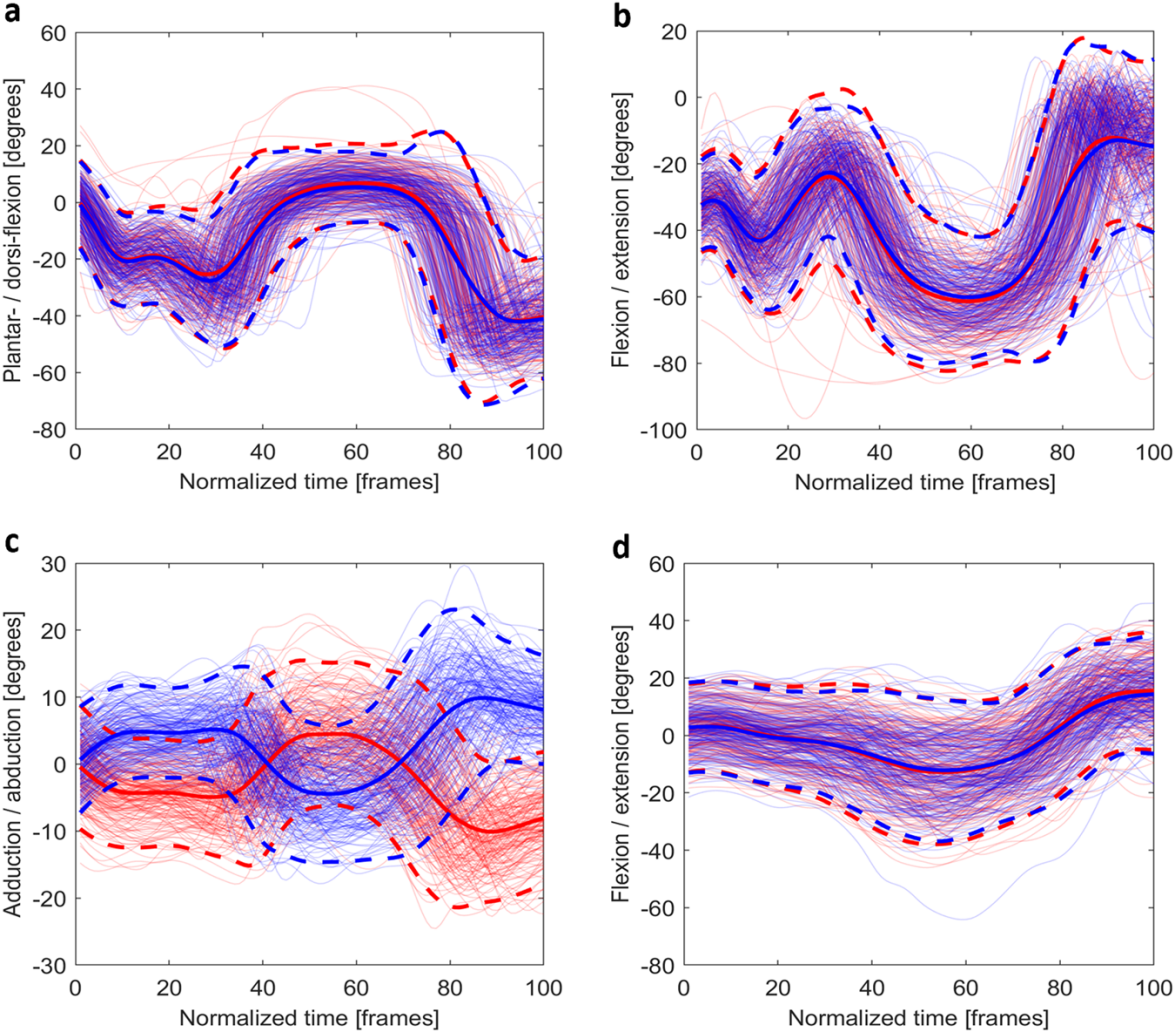
The time normalised joint angles for (**a**) the plantar-/dorsi-flexion (+) of the ankle, (**b**) the flexion/extension (+) of the knee, (**c**) the adduction/abduction (+, left hip; -, right hip) of the hip, and (**d**) the flexion/extension (+) of the trunk during the *hop down left* (blue) and *right* (red). For bilateral joints, the joint presented is the same side as the task. (e.g., for *hop-down left* (blue), the left ankle, knee, and hip are shown). Mean joint angles (solid) and plus/minus two standard deviations (dash) are plotted using opaque lines with the same colour of the joint angles for that side.

**Fig. 5:**
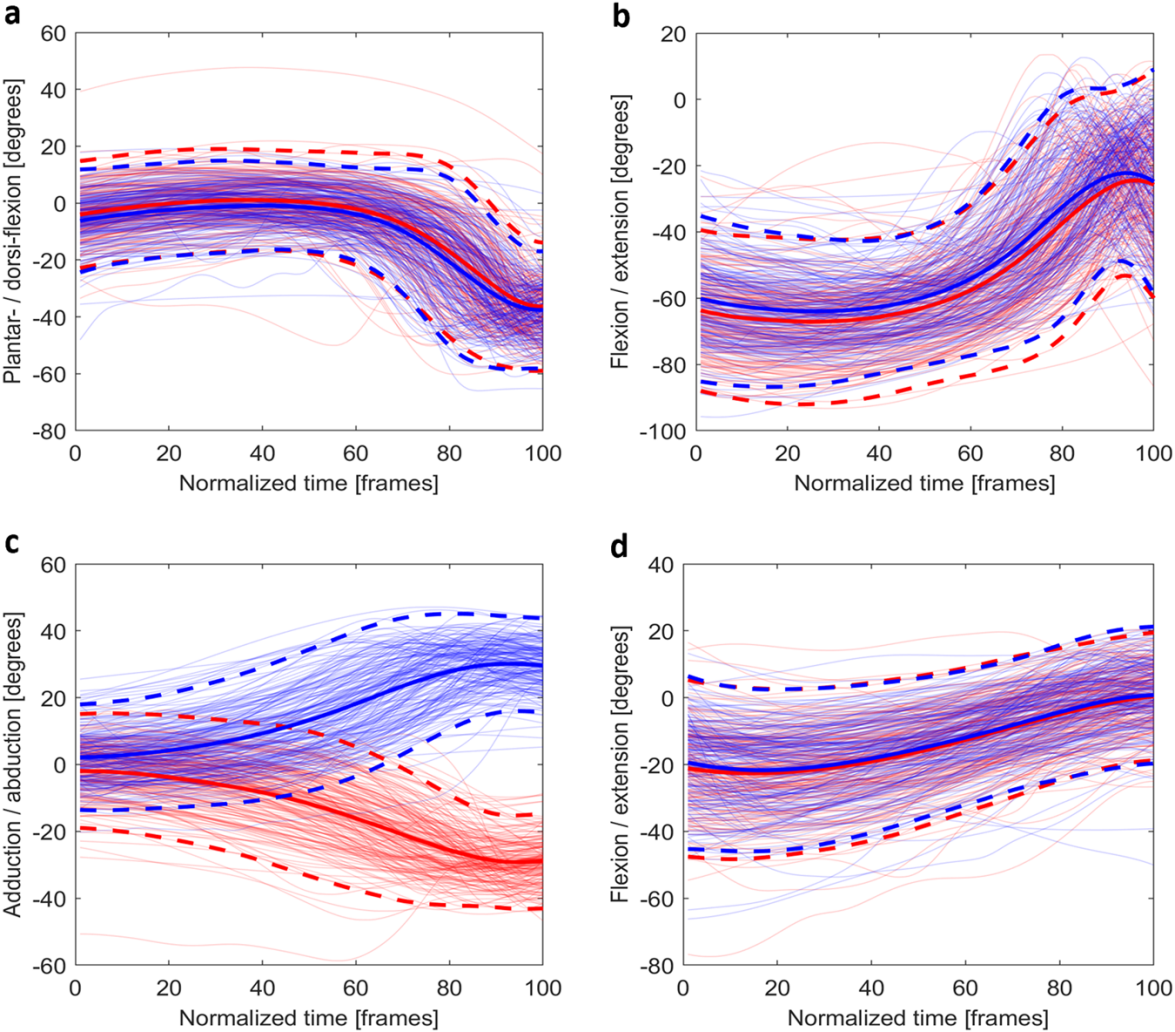
The time normalised joint angles for (**a**) the plantar-/dorsi-flexion (+) of the ankle, (**b**) the flexion/extension (+) of the knee, (**c**) the adduction/abduction (+, left hip; -, right hip) of the hip, and (**d**) the flexion/extension (+) of the trunk during the *L-cut left* (blue) and *right* (red). For bilateral joints, the joint presented is the same side as the task. (e.g., for *L-cut left* (blue), the left ankle, knee, and hips are shown). Mean joint angles (solid) and plus/minus two standard deviations (dash) are plotted using opaque lines with the same colour of the joint angles for that side.

**Fig. 6:**
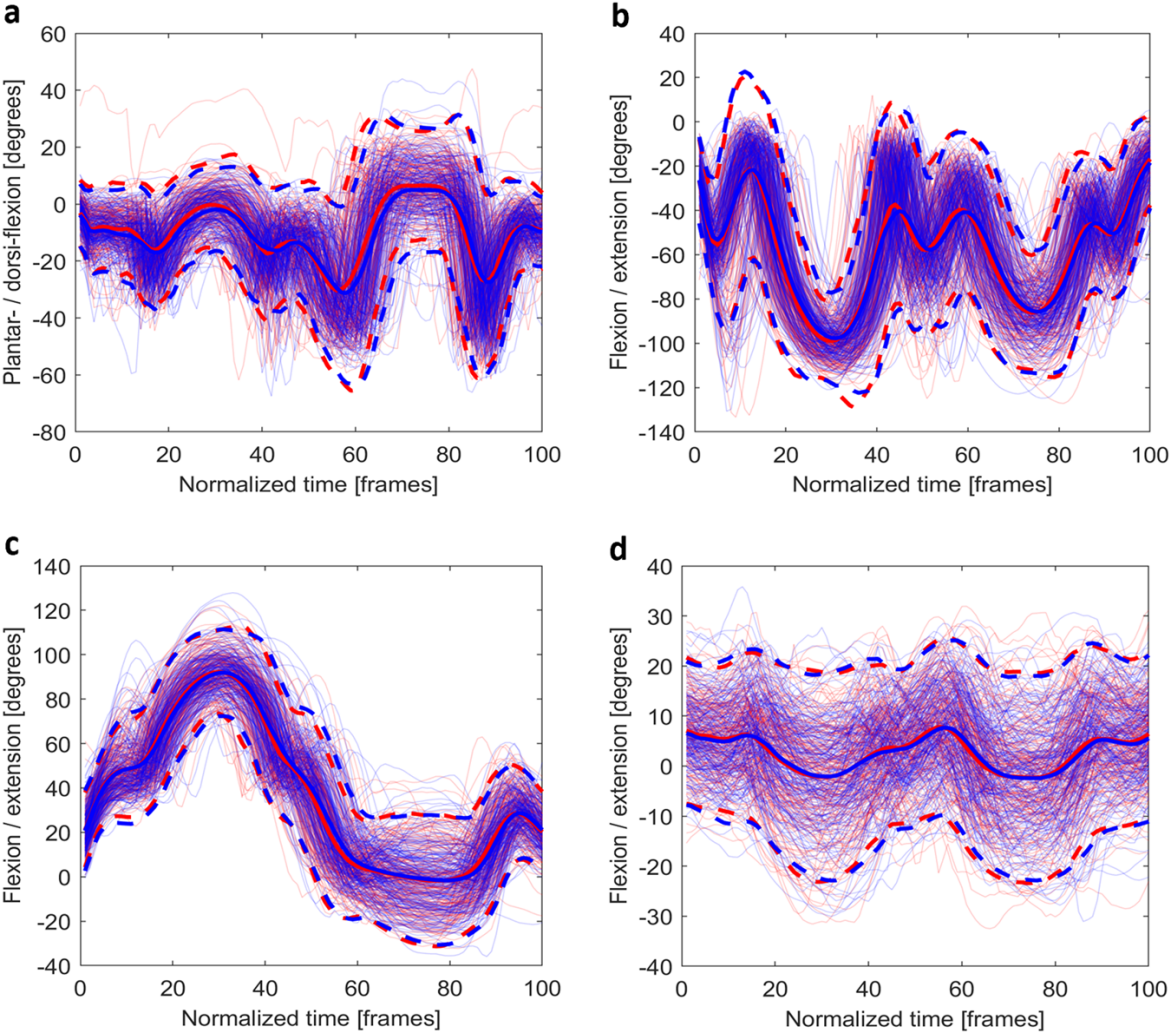
The time normalised joint angles for (**a**) the plantar-/dorsi-flexion (+) of the ankle, (**b**) the flexion/extension (+) of the knee, (**c**) the flexion (+) /extension of the hip, and (**d**) the flexion/extension (+) of the trunk in *Lunge left* (blue) and *right* (red). For bilateral joints, the joint presented is the same side as the task. (e.g., for *Lunge left* (blue), the left ankle, knee, and hip are shown). Mean joint angles (solid) and plus/minus two standard deviations (dash) are plotted using opaque lines with the same colour of the joint angles for that side.

**Fig. 7:**
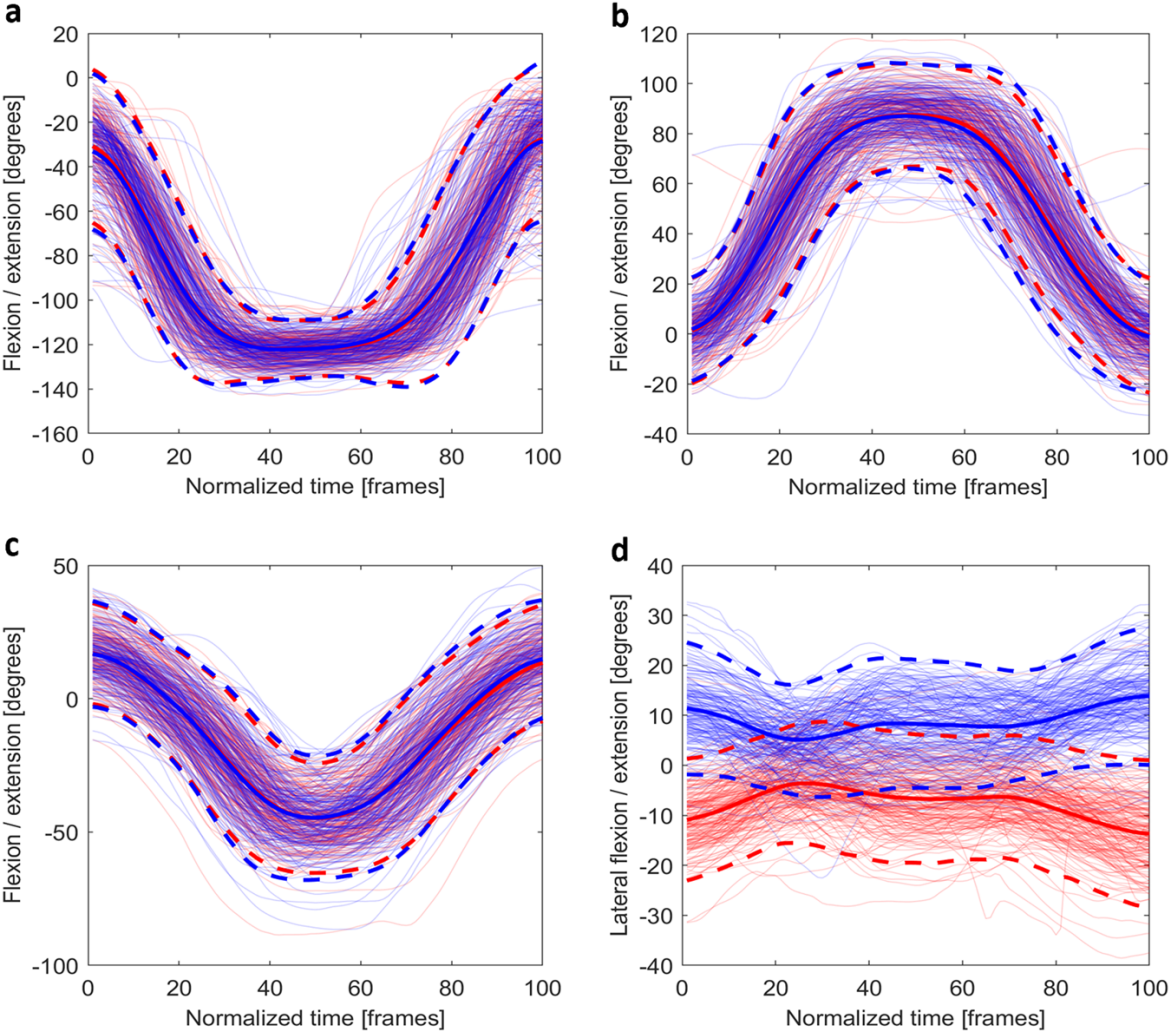
The time normalised joint angles for (**a**) the flexion/extension (+) of the knee, (**b**) the flexion (+) /extension of the hip, (**c**) the flexion/extension (+) of the trunk, and (**d**) the lateral bending (+, left) of the trunk in *rotary stability left* (blue) and *right* (red). For bilateral joints, the joint presented is the opposite side as the task. (e.g., for *rotary stability left* (red), the right knee and hip are shown). Mean joint angles (solid) and plus/minus two standard deviations (dash) are plotted using opaque lines with the same colour of the joint angles for that side.

**Fig. 8:**
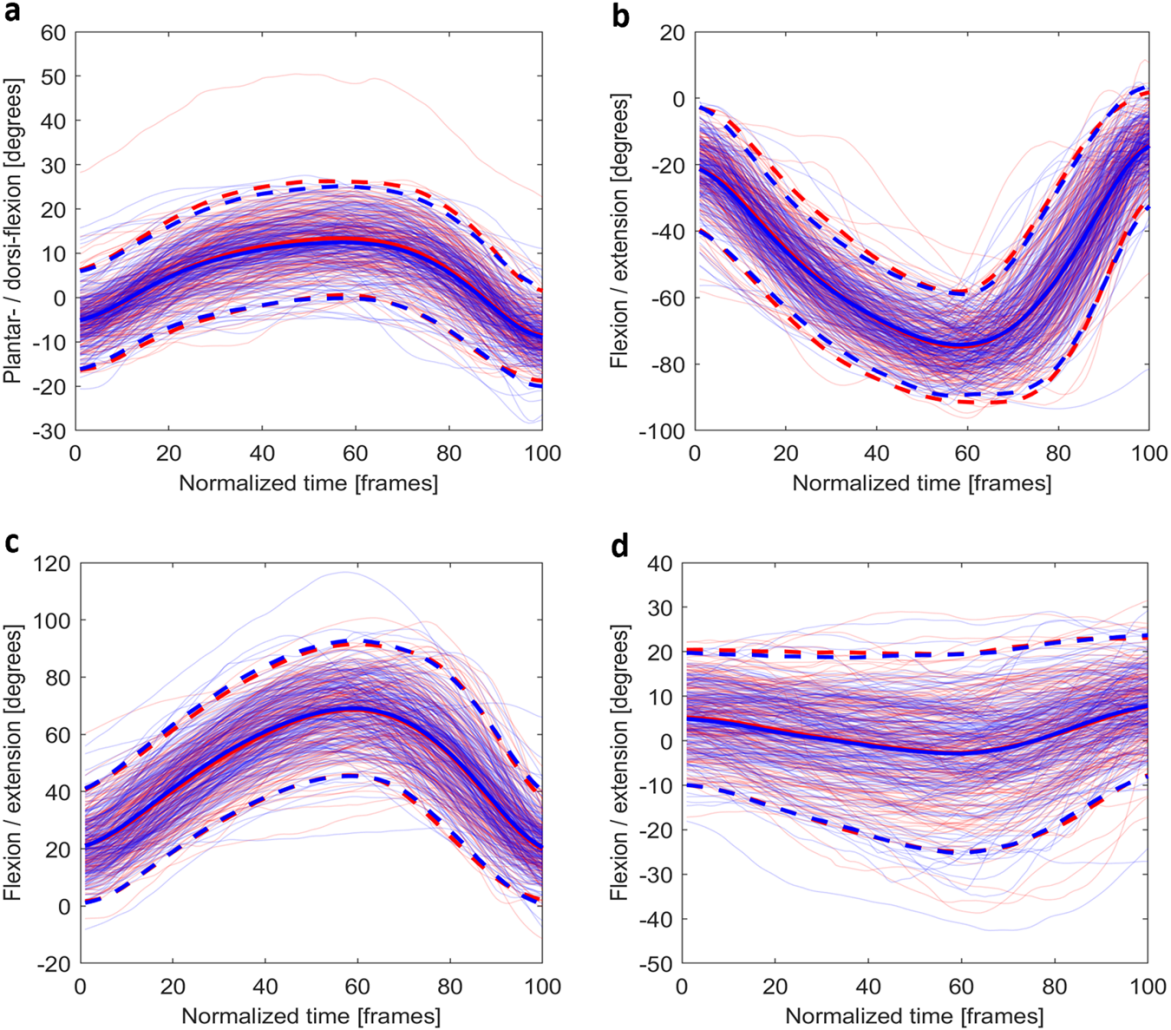
The time normalised joint angles for (**a**) the plantar-/dorsi-flexion (+) of the ankle, (**b**) the flexion/extension (+) of the knee, (**c**) the flexion (+) /extension of the hip, and (**d**) the flexion/extension (+) of the trunk during *step down left* (blue) and *right* (red). For bilateral joints, the joint presented is the same side as the task. (e.g., for *step down left* (blue), the left ankle, knee, and hip are shown). Mean joint angles (solid) and plus/minus two standard deviations (dash) are plotted using opaque lines with the same colour of the joint angles for that side.

**Fig. 9:**
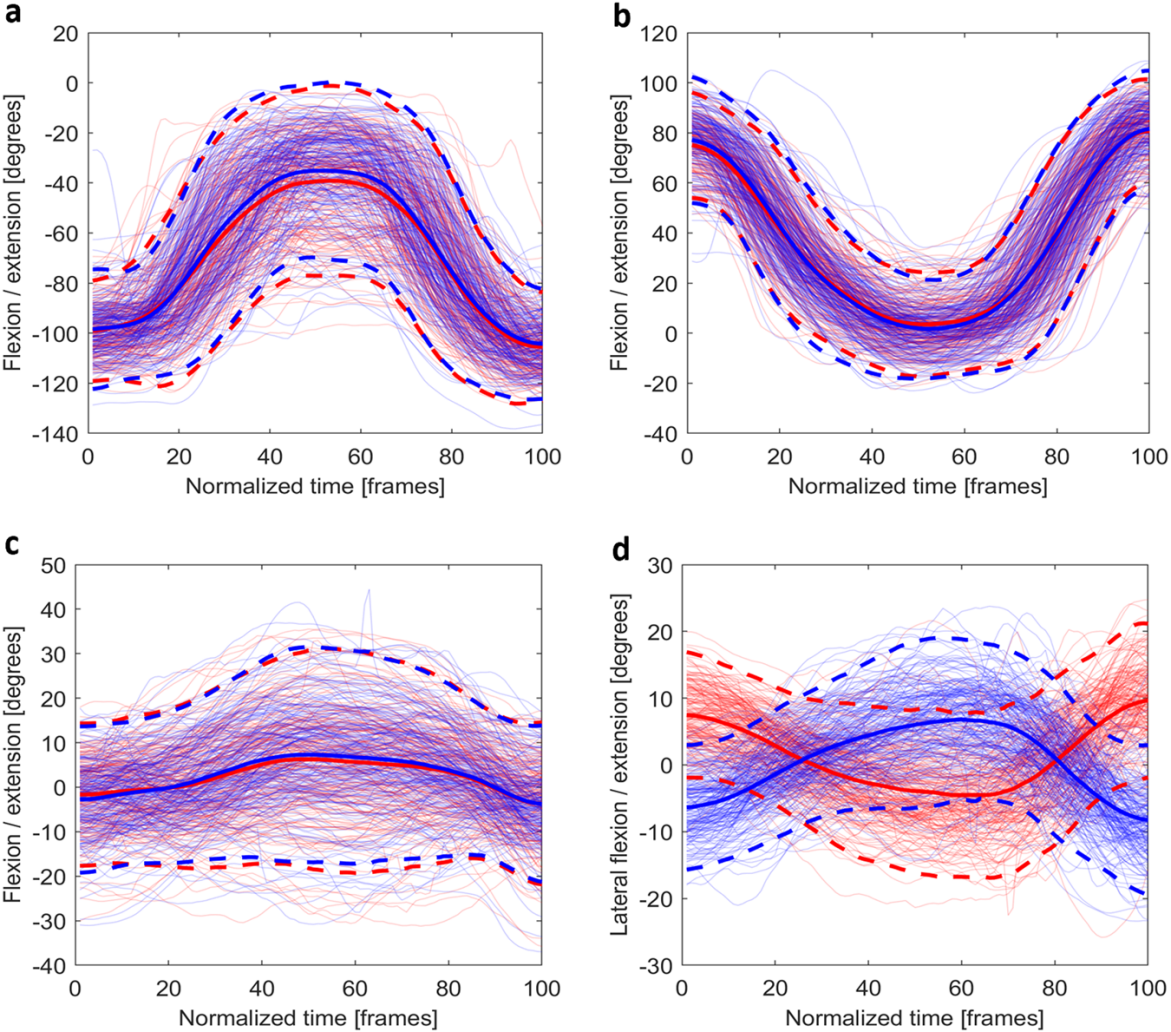
The time normalised joint angles for (**a**) the flexion/extension (+) of the knee, (**b**) the flexion (+) /extension of the hip, (**c**) the flexion/extension (+) of the trunk, and (**d**) the lateral bending (+, left) of the trunk in *T-balance left* (blue) and *right* (red). For bilateral joints, the joint presented is the opposite side as the task. (e.g., for *T-balance left* (red), the right knee and hip are shown). Mean joint angles (solid) and plus/minus two standard deviations (dash) are plotted using opaque lines with the same colour of the joint angles for that side.

Due to the number of tasks included, the seven stability tasks were chosen for validation purposes because they involved multiple joints and planes, rather than the range of motion of a single joint in a single plane. The data were only time-normalised for validation purposes; the provided dataset is not treated with any other normalisation or down-sampling techniques for broader potential applications. For each stability test, the joint angles (*JA_PPID.mat*) in the dominant movement plane (e.g., squat is predominantly performed in the sagittal plane) were selected to validate the data with the custom script. The mean plus/minus two standard deviations (95% confidence interval) of the joint angles across all participants were calculated and compared with published work on the same or similar tasks. Goniometer measured passive ROM was used as a reference if no similar tasks were found. The event indices of each test were used to crop the trials. Since trial lengths were different for each athlete due to athletes self-selecting pace, the data were time-normalised to 101 data points, corresponding to 0–100% of the task, using a Piecewise Cubic Hermite Interpolating Polynomial (PCHIP) spline function *‘pchip’* in Matlab 2019a (Figs. 3–9). Since the *JC_PPID.mat* and *SV_PPID.mat* were calculated from the same model, they are not presented but can be validated in the same fashion.

For the *drop jump* (Fig. 3), *start* and *stop* were used to segment the data. The flexion/extension (or plantar-/dorsi-flexion) and adduction/abduction of the lower limb joint angles were presented. As per previous research, all angles fell within normal ranges^25–27^.

The *hop down* tests were trimmed from *start* to *stop* (Fig. 4). The flexion/extension (or plantar-/dorsi-flexion) of the trunk and lower limb joints and the adduction/abduction of the hip joints were presented. As per previous research, all angles fell within normal ranges^27,28^.

For the *L-cut* (Fig. 5), *landing* and *stop* were selected as the start and the end of this task. During each side of the task, the flexion/extension (or plantar-/dorsi-flexion) of the trunk and the lower limb joints, and the adduction/abduction of the hip joint angles were shown. Despite different protocols, our angles fell within previously reported ranges for cutting movements^29^.

For the *lunge* tests (Fig. 6), the trials were cut based on *start* and *stop*. The flexion/extension angles (or plantar-/dorsi-flexion) of the trunk and lower limbs are shown. According to a study, where only maximal flexion/dorsiflexion at the lower limb joints was reported during a normal forward lunge with the trunk erect, our joint angles fell within the ranges^30^.

For *rotary stability* (Fig. 7), the data were trimmed using *start* and *stop*. The flexion/extension of the hips and knees, and the flexion/extension and lateral bending of the trunk during the tests were selected. All angles fell within normal passive ROMs^26,27^.

For the *step down* (Fig. 8), the flexion/extension (or plantar-/dorsi-flexion) of the ankles, knees, hips, and trunk were trimmed and shown based on *start* and *stop*. All angles fell within normal passive ROMs^26,27^. For plantar flexion, one participant demonstrated a large angle. The participant’s data were reviewed and deemed biofidelic.

For *T-balance* (Fig. 9), the flexion/extension of the knees, hip and trunk, and the lateral bending of the trunk are demonstrated from *start* to *stop* of each trial. During the T-balance, all joint angles were within the range of previously reported passive ROMs^26,27^.

## Usage Notes

The .c3d files can be read using Biomechanical ToolKit (BTK; http://biomechanical-toolkit.github.io/)^31^ or ezc3d (https://github.com/pyomeca/ezc3d), or read and visualized in the Motion Kinematic and Kinetic Analyzer (Mokka; http://biomechanical-toolkit.github.io/mokka/index.html). More detailed information about the file format can be found at https://www.c3d.org. The .mat files can be read and operated in Matlab software (The Mathworks, Inc., Natick, MA, USA). Note that all .c3d files provided in the dataset use their original sampling frequency. Using the default joint angle calculations without IK constraints allows for quadrant flipping (e.g., the shoulder and elbow joint angles jump from +180° to −180° in two consecutive frames). For those interested in upper limb kinematics, it will be important to adjust the model with IK constraints, depending on the specific movement to optimize the calculation for the degree of freedom that is desired.

## Supplementary information

Data checklist

Supplementary File 1

## Data Availability

Python and Matlab scripts used to de-identify the .c3d and .mat files and validate the selected joint angles are available on Github: https://github.com/Graham-Lab1/3D_MoCap_Data_of_a_Movement_Screen. No custom code was used in addition to the Visual3D software to process the dataset.
