# Supplementary material for: Three-Dimensional Motion Capture Data of a Movement Screen from 183 Athletes
Source: Sci Data. 2023 Apr 24;10:235. doi: 10.1038/s41597-023-02082-6 (PMC10126034; doi:10.1038/s41597-023-02082-6)

Supplementary File 1: Movement Description

1.0 Mobility Tests

1.1 Ankle

The subject started on bended knee. With hands at side or resting on thigh, the subject leaned forward, bending at the ankle, until the heel rose. Switched legs and repeated.

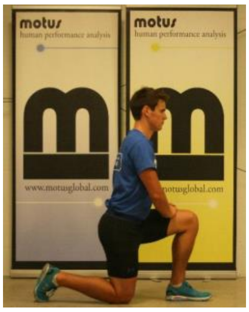

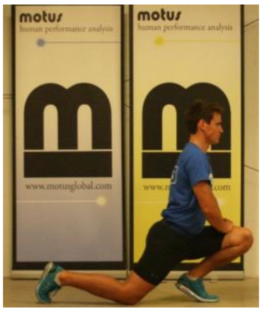

1.2 Back Bend

The subject started in a standing position with feet hip width apart with toes pointed forward. Keeping the knees straight throughout the entire movement, the subject arched their back to end range of motion. This was followed by a forward bend at the waist. If the subject could reach their toes, the arms should then be pulled slightly out and back to allow for maximum pelvic and trunk movement.

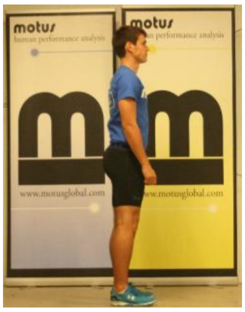

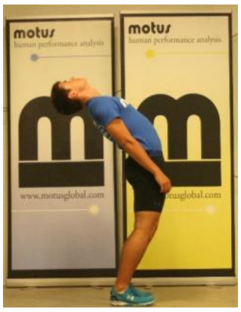

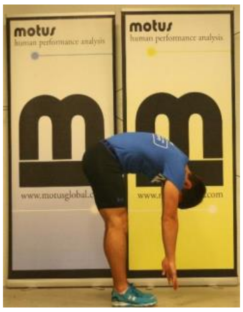

1.3 Crossover Adduction

The subject started in a standing position. Attempting to keep both toes and pelvis facing forward, the subject crossed one leg over the other for maximum distance. The legs should attempt to stay fairly straight, though some bending in the front leg was

tolerable. After reaching a maximally stretched position, the subject continued walking through. Returned to initial standing position and cross legs in opposite direction.

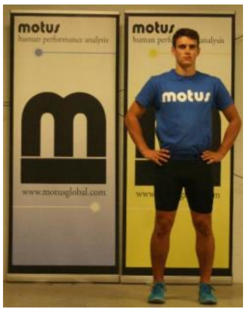

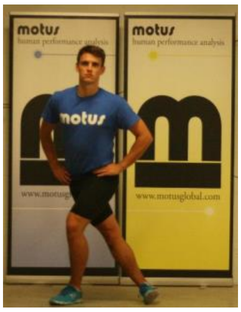

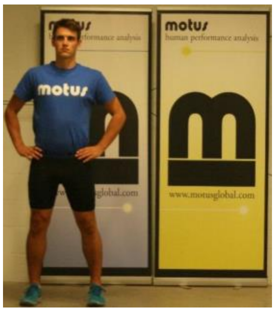

1.4 Crossover Rotation

The subject started in a standing position. Keeping the toe of one foot facing forward and planted on the ground, the pelvis rotated inward and the leg of the opposite foot crossed over. After maximum rotation was achieved, the subject walked through to return to a neutral posture.

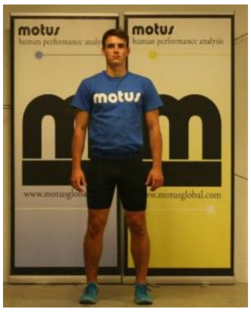

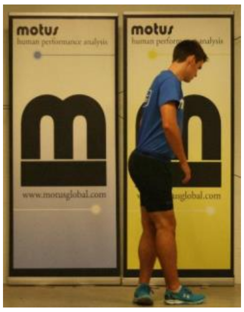

1.5 Elbows

The subject started in a standing position with one arm’s elbow at shoulder height and elbow bent 90° and shoulder rotated so that the hand was near head height and held with a closed fist. The subject straightened their elbow to full extension and then bended to full flexion. Returning to the initial position, the forearm was rotated fully one direction and then the other. Switched arms and repeated.

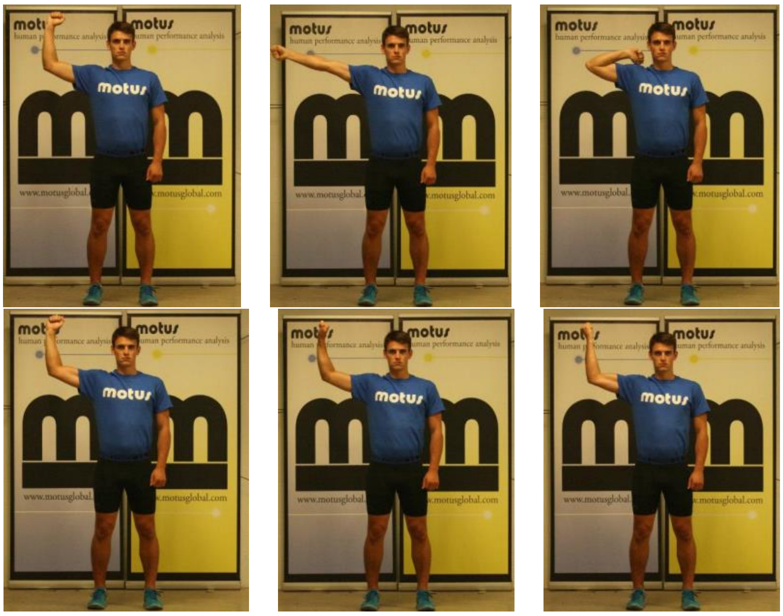

1.6 Head

The subject started in a standing position with head facing forward. The chin was dropped down to the chest and then tilted straight back. The subject returned to the starting position and then rotated the neck so that the chin went towards one shoulder and then counter-rotated towards the other shoulder. Returning again to the starting position, the subject tilted their head, bringing one ear towards the shoulder and then tilted back to bring the other ear to the other shoulder.

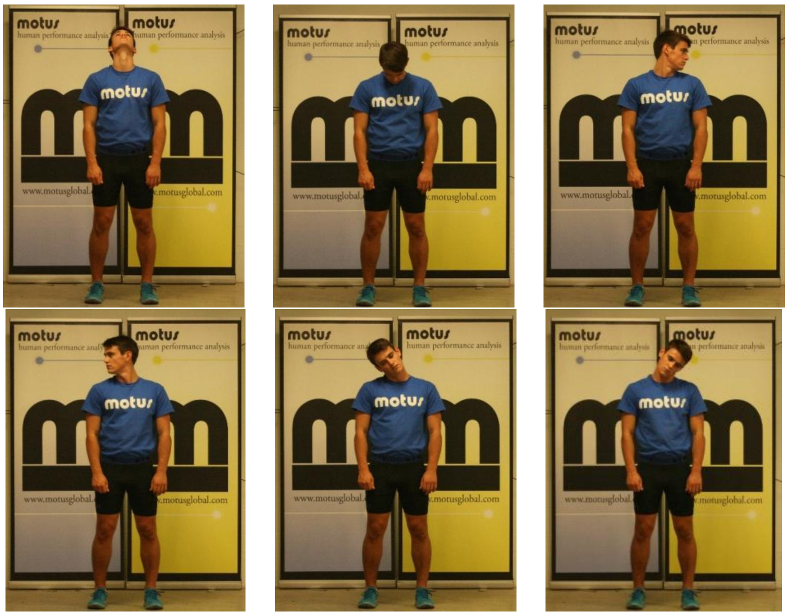

1.7 Hip Turn

The subject started in a standing position with feet hip width apart. With one foot remaining stationary, the subject picked up the other foot slightly off the ground, turned their pelvis open, and maximally rotated on the hip of the planted foot. Once the foot hit the ground, the subject could take a few steps, following their momentum. This was repeated turning the other direction.

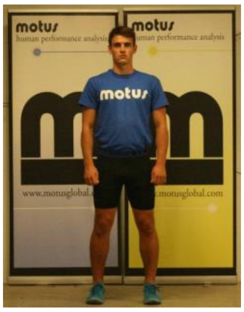

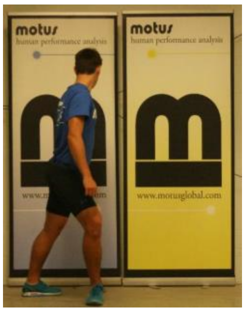

1.8 Scorpion

The subject started in the quadruped position with wrists directly below shoulders and knees directly below hips. Keeping the elbows locked, the back flat, and the abdominals tight, the subject brought one leg back as far as they could, keeping a 90-degree bend in the knee. This was followed by bringing the knee all the way in towards the chest. After returning to the starting position, the action was repeated with the other leg.

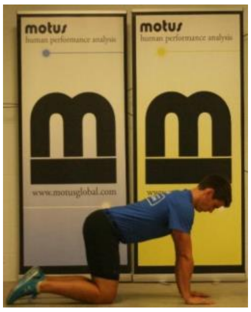

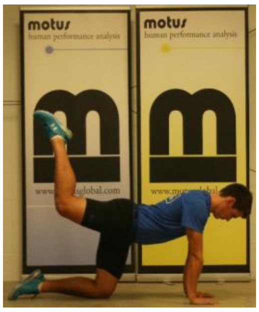

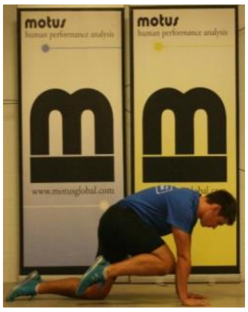

1.9 Shoulder Abduction

The subject started in a standing position with arms at side and elbows bent 90°. Maintaining a bent elbow, the subject lifted the upper arm, bringing the elbow as high as possible while keeping it between the planes of the chest and back. Returned to starting position and repeated with the other arm.

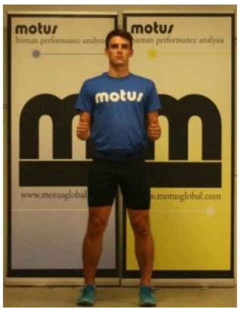

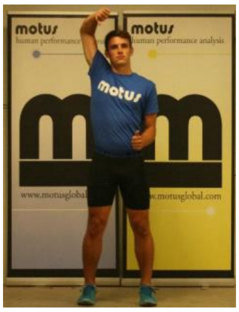

1.11 Shoulder Azimuth

The subject started with one elbow out in line with the shoulders, elbow bent around 90°, and palm facing the ground. Moving at the shoulder while keeping the forearm parallel to the ground and between the height of the chest and shoulders, the subject brought the elbow towards the opposite shoulder. After achieving end range of motion, the subject brought their elbow back in the other direction trying to touch that elbow to the spine. Repeated both actions with opposite arm.

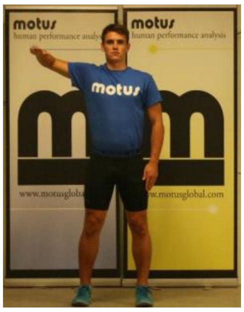

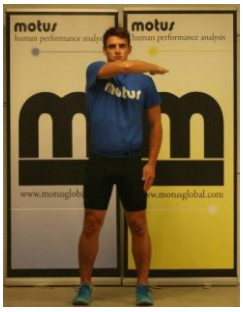

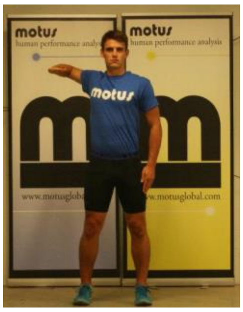

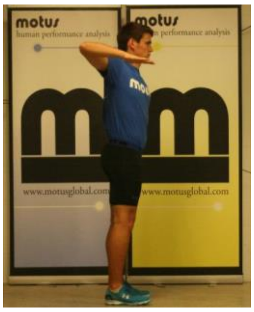

1.12 Shoulder Rotation

The subject started with one elbow out in line with the shoulders, elbow bent around 90°, and palm facing the ground. Keeping the elbow in line with the shoulders, the subject rotated the arm back at the shoulder to end range of motion. The arm was then rotated down at the shoulder to the opposite end range of motion. Repeated both actions with opposite arm.

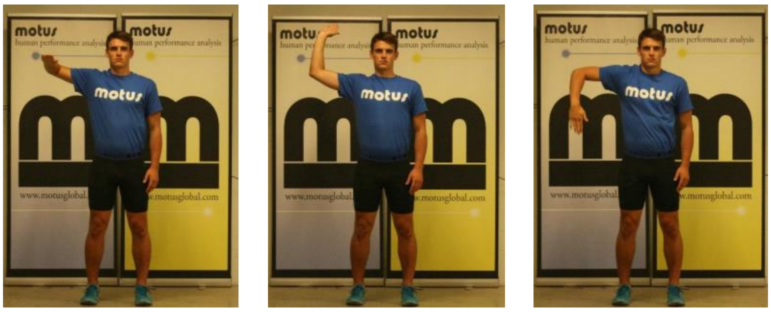

1.13 Side Bends

The subject started in a standing position with arms at sides. Without bending forward at the waist, the subject slid their hand down one leg, bending laterally at the waist. After achieving end range of motion in one direction, the subject returned to the starting position and repeated the action on the other side.

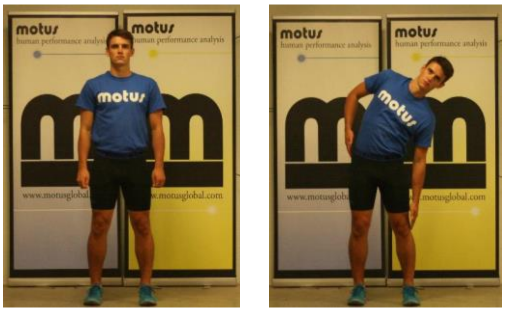

1.14 Side Lunges

The subject started standing with their feet spread out about 80% of maximum distance. Keeping the chest upright and both toes straight and the feet flat on the ground, the subject lunged to one side as they sat back until achieving maximum depth. After returning to center, the subject repeated the action on the other side.

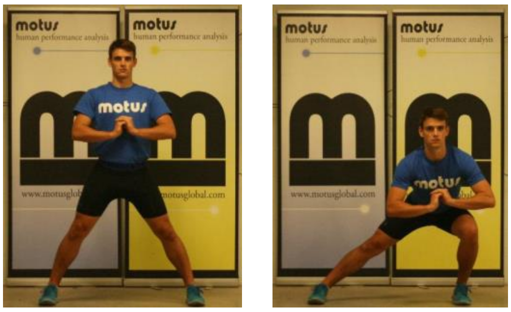

1.15 Trunk Rotation

The subject started in a kneeling position with arms out in front of chest and crossed over one another. Keeping the arms and pelvis still, the subject rotated the upper trunk and chest as far one direction as possible, returned to the starting position, and then rotated the opposite direction.

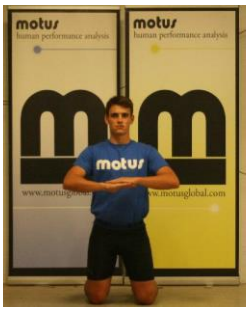

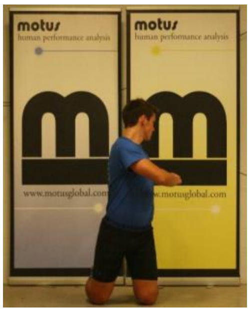

2.0 Stability Tests

2.1 Drop Jump

The subject started by standing on a 30 cm tall platform. The subject then dropped down off the platform to the floor and upon landing immediately transitioned into a maximal vertical height jump. Arm swing was encouraged to help achieve maximal height.

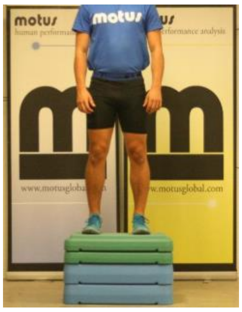

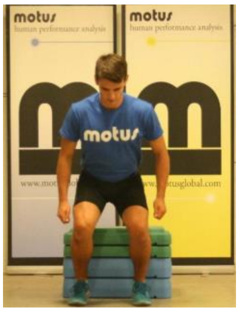

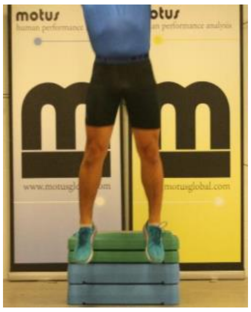

2.2 Hop Down

The subject started on a box approximately 15-30 cm high (depending on subject's height). Standing on one foot, the subject hopped off the box, landed on that same foot, and immediately performed a maximum vertical jump with arms up. Arm swing was encouraged to help achieve maximal height. Test was repeated on opposite leg.

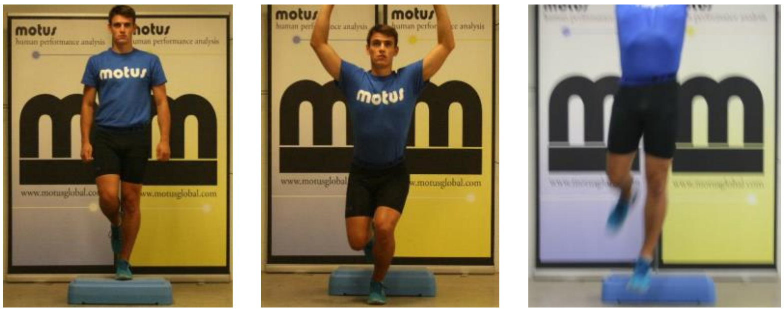

2.3 L-Cut

The subject started in a standing position with feet hip width apart. The subject jumped as far forward as they could, landing on their left foot. Immediately after the foot touched the ground, the subject cut 90° to the right side, landing on both feet. The L-cut was then repeated with the other leg.

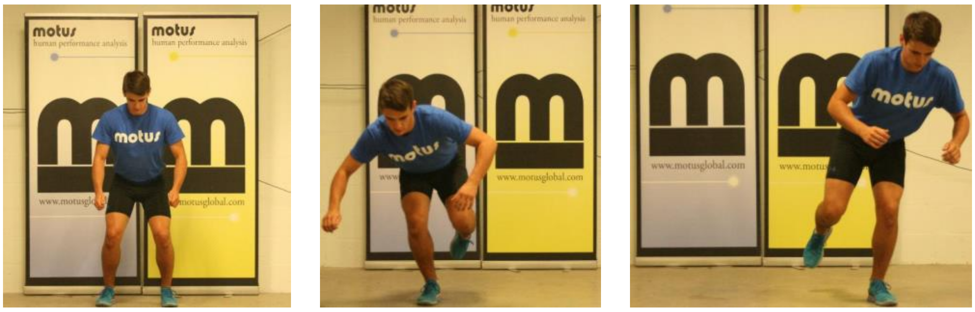

2.4 Lunge

The subject started in a standing position with feet hip width apart and hands on hips. The subject lunged forward a distance equal to their leg's length (measured from the greater trochanter to the floor). The depth should be close to the ground or when the knee gently touched the ground. After reaching this depth, the subject stepped back in a backward lunge. The lunge length and depth should be the same as the forward lunge. The subject was not permitted to touch the ground with the foot of the lunging leg at any point during the transition from forward to backward. They must also keep their head facing forward throughout the entire movement. After completing the backward lunge, the subject returned to their initial standing position. The forward and backward lunges were then repeated with the other leg.

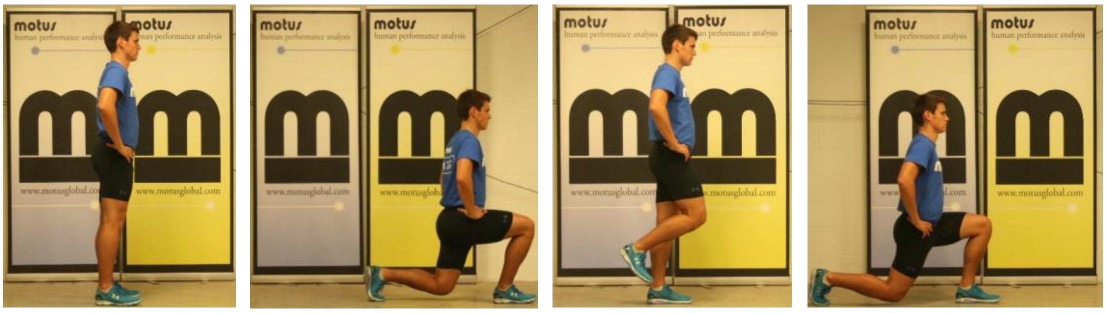

2.5 Rotary Stability

The subject started in the quadruped position with hands directly below shoulders and knees directly below hips. Maintaining a flat back, the subject lifted and straightened one arm and the opposite leg to a height level with the back. After holding this position for a three-count, the subject brought their elbow of the raised arm in as they brought in their knee of the raised leg to a tucked position and touched them. The subject returned to the initial position with arm and opposite leg extended and held for another three-count. This action was repeated raising the other arm and opposite leg.

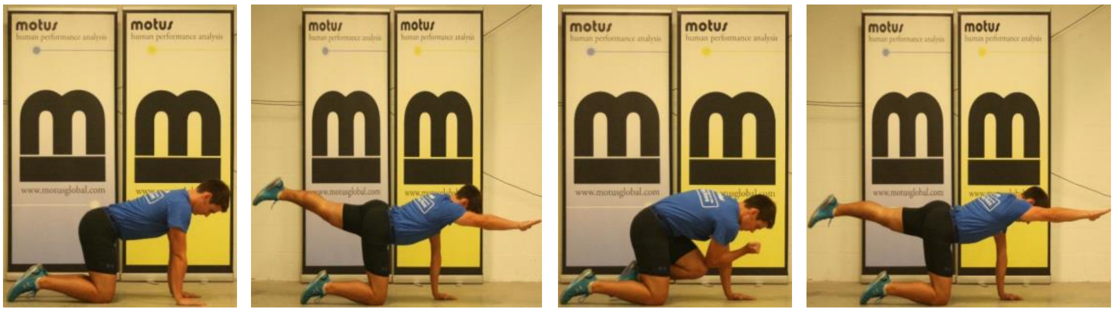

2.6 Step Down

The subject started standing with one foot on a box approximately the height from the floor to their mid-shank. The foot of the other leg should hang off the edge with the toes pointed slightly upward and the heel in line with the mid-foot of the planted leg. With hands in a "prayer position" in front of the chest, the subject squatted down in a slow, controlled manner until the heel of the opposite leg tapped the ground. Immediately after that heel tapped the grounds, the subject returned to the initial standing position. This action was then repeated with the other leg.

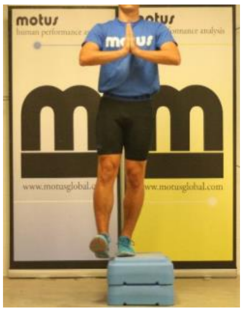 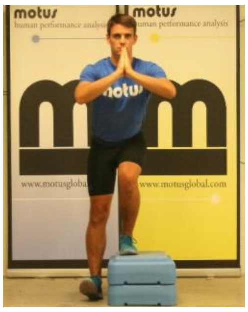

2.7 T-Balance

The subject started in a standing position. Bringing the hands in front of the chest in the “prayer position, the subject lifted one leg so that the thigh was roughly parallel to the ground. After holding that position for a three-count, the subject began leaning forward at the waist, straightening their arms and swing leg to create a “T” with the three limbs. The subject should attempt to achieve maximal forward bending at the waist without losing balance. Ideally, the plant leg should remain as straight as possible, but some bending is permitted. As soon as the subject achieved their forward, most rotated position, they returned to the initial position with their hands in the prayer position and the thigh of the swing leg roughly parallel to the ground. This position was again held for a three-count. The movement was then repeated while balancing on the opposite leg.

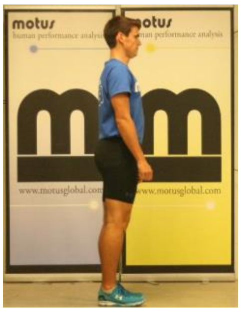 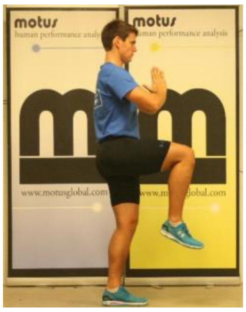 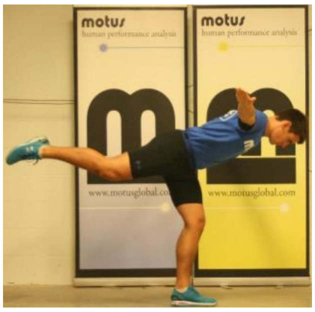

Supplement: Supplementary file 2 — Supplementary File 1 [file 41597_2023_2082_MOESM2_ESM.pdf]
